# Practice of stress management behaviors and associated factors among undergraduate students of Mekelle University, Ethiopia: a cross-sectional study

**DOI:** 10.1186/s12888-020-02574-4

**Published:** 2020-04-15

**Authors:** Gebrezabher Niguse Hailu

**Affiliations:** School of nursing, Mekelle University, Tigray, Ethiopia

**Keywords:** Stress management, Behaviors, University students, Ethiopia

## Abstract

**Background:**

Stress is one of the top five threats to academic performance among college students globally. Consequently, students decrease in academic performance, learning ability and retention. However, no study has assessed the practice of stress management behaviors and associated factors among college students in Ethiopia. So the purpose of this study was to assess the practice of stress management behaviors and associated factors among undergraduate university students at Mekelle University, Tigray, Ethiopia, 2019.

**Methods:**

A cross-sectional study was conducted on 633 study participants at Mekelle University from November 2018 to July 2019. Bivariate analysis was used to determine the association between the independent variable and the outcome variable at *p* < 0.25 significance level. Significant variables were selected for multivariate analysis.

**Results:**

The study found that the practice of stress management behaviors among undergraduate Mekelle university students was found as 367(58%) poor and 266(42%) good. The study also indicated that sex, year of education, monthly income, self-efficacy status, and social support status were significant predictors of stress management behaviors of college students.

**Conclusion:**

This study found that the majority of the students had poor practice of stress management behaviors.

## Background

Stress is the physical and emotional adaptive response to an external situation that results in physical, psychological and behavioral deviations [1]. Stress can be roughly subdivided into the effects and mechanisms of chronic and acute stress [2]. Chronic psychological stress in early life and adulthood has been demonstrated to result in maladaptive changes in both the HPA-axis and the sympathetic nervous system. Acute and time-limited stressors seem to result in adaptive redistribution of all major leukocyte subpopulations [2].

Stress management behaviors are defined as behaviors people often use in the face of stress /or trauma to help manage painful or difficult emotions [3]. Stress management behaviors include sleeping 6–8 h each night, Make an effort to monitor emotional changes, Use adequate responses to unreasonable issues, Make schedules and set priorities, Make an effort to determine the source of each stress that occurs, Make an effort to spend time daily for muscle relaxation, Concentrate on pleasant thoughts at bedtime, Feel content and peace with yourself [4]. Practicing those behaviors are very important in helping people adjust to stressful events while helping them maintain their emotional wellbeing [3].

University students are a special group of people that are enduring a critical transitory period in which they are going from adolescence to adulthood and can be one of the most stressful times in a person’s life [5]. According to the American College Health Association’s National College Health Assessment, stress is one of the top five threats to academic performance among college students [6]. For instance, stress is a serious problem in college student populations across the United States [7].

I have searched literatures regarding stress among college students worldwide. For instance, among Malaysian university students, stress was observed among 36% of the respondents [8]. Another study reported that 43% of Hong Kong students were suffered from academic stress [9]. In western countries and other Middle Eastern countries, including 70% in Jordan [10], 83.9% in Australia [11]. Furthermore, based on a large nationally representative study the prevalence of stress among college students in Ethiopia was 40.9% [12].

Several studies have shown that socio-demographic characteristics and psychosocial factors like social support, health value and perceived self-efficacy were known to predict stress management behaviors [13–17].

Although the prevalence of stress among college students is studied in many countries including Ethiopia, the practice of stress management behaviors which is very important in promoting the health of college students is not studied in Ethiopia. Therefore this study aimed to assess the practice of stress management behaviors and associated factors among undergraduate students at Mekelle University.

## Methods

The study was conducted at Mekelle university colleges from November 2018 to July 2019 in Mekelle city, Tigray, Ethiopia. Mekelle University is a higher education and training public institution located in Mekelle city, Tigray at a distance of 783 Kilometers from the Ethiopian capital (http://www.mu.edu.et/).

A cross-sectional study was conducted on 633 study participants. Students who were ill (unable to attend class due to illness), infield work and withdrawal were not included in the study.

The actual sample size (n) was computed by single population proportion formula [n = [(Za/2)2*P (1 − P)]/d2] by assuming 95% confidence level of Za/2 = 1.96, margin of error 5%, proportion (p) of 50% and the final sample size was estimated to be 633. A 1.5 design effect was used by considering the multistage sampling technique and assuming that there was no as such big variations among the students included in the study.

Multi-stage random sampling was used. Three colleges (College of health science, college of business and Economics and College of Natural and Computational Science) were selected from a total of the seven Colleges from Mekelle University using a simple random sampling technique in which proportional sample allocation was considered from each college.

Data were collected using a self-administered questionnaire by trained research assistants at the classes.

The questionnaire has three sections. The first section contained questions on demographic characteristics of the study participants. The second section contained questions to assess the practice of stress management of the students. The tool to assess the practice of stress management behaviors for college students was developed by Walker, Sechrist, and Pender [4]. The third section consisted of questions for factors associated with stress management of the students divided into four sub-domains, including health value used to assess the value participants place on their health [18]. The second subdomain is self-efficacy designed to assess optimistic self-beliefs to cope with a variety of difficult demands in life [19] and was adapted by Yesilay et al. [20]. The third subdomain is perceived social support measures three sources of support: family, friends, and significant others [21] and was adapted by Eker et al. [22]. The fourth subscale is perceived stress measures respondents’ evaluation of the stressfulness of situations in the past month of their lives [23] and was adapted by Örücü and Demir [24].

The entered data were edited, checked visually for its completeness and the response was coded and entered by Epi-data manager version 4.2 for windows and exported to SPSS version 21.0 for statistical analysis.

Bivariate analysis was used to determine the association between the independent variable and the outcome variable. Variables that were significant at *p* < 0.25 with the outcome variable were selected for multivariable analysis. And odds ratio with 95% confidence level was computed and *p*-value <= 0.05 was described as a significant association.

### Operational definition

#### Good stress management behavior:

Students score above or equal to the mean score.

#### Poor stress management behavior:

Students score below the mean score [4].

## Results

### Seciodemographic characteristics

Among the total 633 study participants, 389(61.5%) were males, of those 204(32.2%) had poor stress management behavior. The Median age of the respondents was 20.00 (IQR = ±3). More ever, this result showed that 320(50.6%) of the students came from rural areas, 215(34%) of them had poor stress management behavior.

The result revealed that 363(57.35%) of the study participants were 2nd and 3rd year students, of them 195 (30.8%) had poor stress management.

This result indicated that 502 (79.3%) of the participants were in the monthly support category of > = 300 ETB with a median income of 300.00 ETB (IQR = ±500), from those, 273(43.1%) students had poor stress management behavior (Table 1).

**Table 1:** Distribution of socio-demographic characteristics and psychosocial factors of undergraduate students with the practice of stress management behaviors, 2019 (*n* = 633)

Variable	Category	Practice of stress management behaviors		Total n (%)
		Poor n (%)	Good n (%)	
Sex	Female	163(25.75)	81(12.8)	244(38.6)
	Male	204(32.2)	185(29.23)	389(61.5)
Age	> = 20 year	204(32.2)	244(38.55)	448(70.8)
	< 20 years (18-19)	163(25.75)	22(3.5)	185(2.23)
College	CHS	101(16)	167(26.4)	268(42.0)
	CBE	116(18.33)	40(6.3)	156(24.6)
	CNCS	150(23.7)	59(9.3)	209(33)
Year of education	4th year	30(4.74)	36(5.7)	66(10.4)
	2nd & 3rd year	195(30.81)	168(26.5)	363(56.7)
	1st year	171(27)	33(5.2)	204(32.23)
Parent’s resident	Urban	152(24)	161(25.4)	313(49.45)
	Rural	215(34)	105(16.6)	320(50.6)
Student’s monthly income (ETB)	> = 300 ETB	273(43.1)	229(36.2)	502(79.3)
	< 300ETB	94(14.85)	37(5.8)	131(20.7)
Psychosocial Factors				
Health value status	High	137(21.6)	215(34)	352(55.61)
	Low	230(36.3)	51(8.1)	281(44.4)
Perceived self -efficacy	Good	230(36.3)	241(38.1)	471(74.41)
	Poor	131(20.7)	31(4.9)	162(25.6)
Perceived social support	Good	133(21)	150(23.7)	201(31.8)
	Poor	234(37)	116(18.33)	432(68.2)
perceived stress status	yes	127(20.1)	158(25)	285(45)
	No	240(37.9)	108(17.1)	318(50.2)

**Fig. 1:**
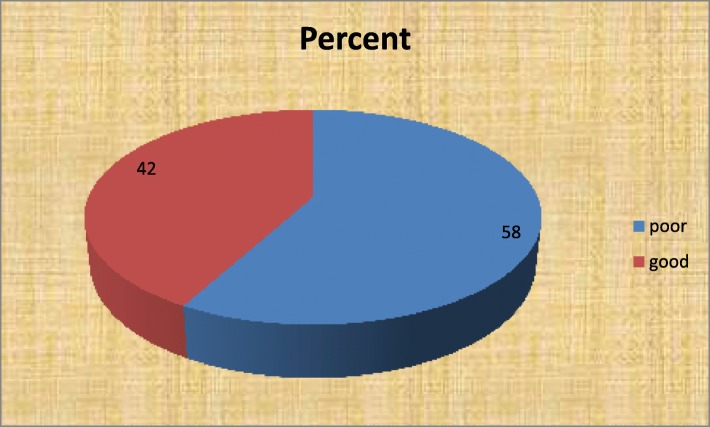
Status of practice of stress management behaviors of under graduate students at Mekelle University, Ethiopia

### Psychosocial factors

This result indicated that 352 (55.6%) of the students had a high health value status of them 215 (34%) had good stress management behavior. It also showed that 162 (25.6%) of the students had poor perceived self-efficacy, from those 31(4.9%) had a good practice of stress management behavior. Moreover, the result showed that 432(68.2%) of the study participants had poor social support status of them 116(18.3%) had a good practice of stress management behavior (Table 1).

### Practice of stress management behaviors

The result showed that the majority (49.8%) of the students were sometimes made an effort to spend time daily for muscle relaxation. Whereas only 28(4.4%) students were routinely concentrated on pleasant thoughts at bedtime.

According to this result, only 169(26.7%) of the students were often made an effort to determine the source of stress that occurs. It also revealed that the majority (40.1%) of the students were never made an effort to monitor their emotional changes. Similarly, the result indicated that the majority (42.5%) of the students were never made schedules and set priorities.

The result revealed that only 68(10.7%) of the students routinely slept 6–8 h each night. More ever, the result showed that the majority (34.4%) of the students were sometimes used adequate responses to unreasonable issues (Table 2).

**Table 2:** Distribution of practice of stress management behaviors of undergraduate students, 2019 (*n* = 633)

Items	Never	Sometimes	Often	Routinely
Make an effort to spend time daily for muscle relaxation	192(30.3)	315(49.77)	63(9.95)	63(9.95)
Concentrate on pleasant thoughts at bedtime	117(18.2)	302(47.71)	126(19.91)	28(4.4)
Feel content and peace with myself	212(33.5)	200(31.6)	174(27.49)	47(7.4)
Make an effort to determine the source of each stress that occurs	170(26.86)	238(37.6)	169(26.7)	56(8.85)
Make an effort to monitor my emotional changes	254(40.1)	190(30)	126(19.91)	63(9.95)
Sleep 6–8 h. each night	152(24)	217(34.28)	196(30.97)	68(10.74)
Make schedules and set priorities	269(42.5)	206(32.5)	140(22.1)	18(2.84)
Use adequate responses to unreasonable issues	206(32.54)	218(34.44)	148(23.38)	61(9.64)

### Status of the practice of stress management behaviors

The result revealed that the practice of stress management behaviors among regular undergraduate Mekelle university students was found as 367(58%) poor and 266(42%) good. (Fig 1)

### Factors associated with stress management behaviors

In the bivariate analysis sex, college, year of education, student’s monthly income’, perceived-self efficacy, perceived social support and perceived stress were significantly associated with stress management behavior at p < =0.25. Whereas in the multivariate analysis sex, year of education, student’s monthly income’, perceived-self efficacy and perceived social support were significantly associated with stress management behavior at p < =0.05.

Male students were 3.244 times more likely to have good practice stress management behaviors than female students (AOR: 3.244, CI: [1.934–5.439]). Students who were in the age category of less than 20 years were 70% less to have a good practice of stress management behaviors than students with the age of greater or equal to 20 year (AOR: 0.300, CI:[0.146–0.618]).

Students who had monthly income less than300 ETB were 64.4% less to have a good practice of stress management behaviors than students with monthly income greater or equal to 300 ETB (AOR: 0.356, CI:[0.187–0.678]).

Students who had poor self- efficacy status were 70.3% less to have a good practice of stress management behaviors than students with good self-efficacy status (AOR: 0.297, CI:[0.159–0.554]). Students who had poor social support were 70.5% less to have a good practice of stress management behaviors than students with good social support status (AOR: 0.295[0.155–0.560]) (Table 3).

**Table 3:** Bivariate and multivariable results of factors associated with practice of stress management behaviors of undergraduate Mekelle university students, 2019 (*n* = 633)

Variable	Category	practice of Stress management behaviors		*P*-value	COR	AOR [CI]
		Poor	Good			
Sex	Female	163	81			1
	Male	204	185	**0.000**	1.825	3.244[1.934–5.439]
Age	> = 20 year	204	244			
	< 20 years (18-19)	163	22	0.520	0.113	
Year of education	4th year	30	36			1
	2nd & 3rd year	195	168	0.062	0.720	
	1st year	171	33	**0.000**	0.161	0.012 [0.001–0.120]
		116	40			
Student’s income (ETB)	> = 300ETB	273	229			1
	< 300 ETB	94	37	**0.002**	0.469	0.356[0.187–0.678]
College	CHS	101	167			1
	CBE	116	40	0.210	0.209	
	CNCS	150	59	0.056	0.238	
Parent’s resident	Urban	152	161			1
	Rural	215	105	0.256	0.461	
Health value status	High	137	215			
	Low	230	51	0.301	0.141	
Perceived self- efficacy	Good	230	241			
	Poor	131	31	**0.000**	0.226	0.297[0.159–0.554]
Perceived social support	Good	133	150			1
	Poor	234	116	**0.000**	0.440	0.295[0.155–0.560]
Perceived stress status	yes	127	158			1
	No	240	108	0.571	0.344	

## Discussion

The present study showed that the practice of stress management behaviors among regular undergraduate students was 367(58%) poor and 266(42%) good. The study indicated that sex, year of education, student’s monthly income, social support status, and perceived-self efficacy status were significant predictors of stress management behaviors of students.

The current study revealed that male students were more likely to have good practice of stress management behaviors than female students. This finding is contradictory with previous studies conducted in the USA [13, 25], where female students were showed better practice of stress management behaviors than male students. This difference might be due to socioeconomic and measurement tool differences.

The current study indicated that students with monthly income less than 300 ETB were less likely to have good practice of stress management behaviors than students with monthly income greater than or equal to 300 ETB. This is congruent with the recently published book which argues a better understanding of our relationship with money (income). The book said “the people with more money are, on average, happier than the people with less money. They have less to worry about because they are not worried about where they are going to get food or money for their accommodation or whatever the following week, and this has a positive effect on their health” [26].

The present study found that first-year students were less likely to have good practice of stress management behaviors than senior students. This finding is similar to previous findings from Japan [27], China [28] and Ghana [29]. This might be because freshman students may encounter a multitude of stressors, some of which they may have dealt with in high school and others that may be a new experience for them. With so many new experiences, responsibilities, social settings, and demands on their time. As a first-time, incoming college freshman, experiencing life as an adult and acclimating to the numerous and varied types of demands placed on them can be a truly overwhelming experience. It can also lead to unhealthy amounts of stress. A report by the Anxiety and Depression Association of America found that 80% of freshman students frequently or sometimes experience daily stress [30].

The current study showed that students with poor self-efficacy status were less likely to have good practice of stress management behaviors. This is congruent with the previous study that has demonstrated quite convincingly that possessing high levels of self-efficacy acts to decrease people’s potential for experiencing negative stress feelings by increasing their sense of being in control of the situations they encounter [14]. More ever this study found that students with poor social support were less likely to have a good practice of stress management behaviors. This finding is similar to previous studies that found good social support, whether from a trusted group or valued individual, has shown to reduce the psychological and physiological consequences of stress, and may enhance immune function [15–17].

### Ethics approval and consent to participate

Ethical clearance and approval obtained from the institutional review board of Mekelle University. Moreover, before conducting the study, the purpose and objective of the study were described to the study participants and written informed consent was obtained. The study participants were informed as they have full right to discontinue during the interview. Subject confidentiality and any special data security requirements were maintained and assured by not exposing the patient’s name and information.

### Limitation of the study

There is limited literature regarding stress management behaviors and associated factors. There is no similar study done in Ethiopia previously. More ever, using a self-administered questionnaire, the respondents might not pay full attention to it/read it properly.

## Conclusion

This study found that the majority of the students had poor practice of stress management behaviors. The study also found that sex, year of education, student’s monthly income, social support status, and perceived-self efficacy status were significant predictors of stress management behaviors of the students.

## Data Availability

The datasets used during the current study is available from the corresponding author on reasonable request.

## Abbreviations

AOR: Adjusted Odd Ratio
CBE: College of Business& Economics
CHS: College of health sciences
CI: Confidence interval
CNCS: College of natural and computational sciences
COR: Crud odds ratio
ETB: Ethiopian birr
MSC: Master of Sciences
USA: United States of America
UK: United kingdom
